# *TNFAIP3* mutation is an independent poor overall survival factor for patients with T‐cell acute lymphoblastic leukemia

**DOI:** 10.1002/cam4.5196

**Published:** 2022-09-03

**Authors:** Cunte Chen, Lingling Zhou, Lihua Zhu, Gengxin Luo, Liang Wang, Chengwu Zeng, Hongsheng Zhou, Yangqiu Li

**Affiliations:** ^1^ Institute of Hematology, School of Medicine, Key Laboratory for Regenerative Medicine of Ministry of Education Jinan University Guangzhou China; ^2^ Department of Hematology, Nanfang Hospital Southern Medical University Guangzhou China; ^3^ Department of Rheumatism and Immunology, First Affiliated Hospital Jinan University Guangzhou China; ^4^ Department of Hematology, First Affiliated Hospital Jinan University Guangzhou China; ^5^ Department of Oncology, First Affiliated Hospital Jinan University Guangzhou China

**Keywords:** biomarker, prognosis, T‐cell acute lymphoblastic leukemia, TNFAIP3 mutation

## Abstract

**Background:**

It is imperative to explore potential biomarkers for predicting clinical outcome and developing targeted therapies for T‐cell acute lymphoblastic leukemia (T‐ALL). This study aimed to investigate the mutation patterns of tumor necrosis factor‐alpha‐inducing protein 3 (*TNFAIP3*, also known as *A20*) and its role in the prognosis of T‐ALL patients.

**Methods:**

Polymerase chain reaction (PCR) and Sanger sequencing data from T‐ALL (*n* = 49, JNU) and targeted sequencing data from T‐ALL (*n* = 54, NFH) in our clinical center and a publicly available dataset (*n* = 121, PRJCA002270), were used to detect *TNFAIP3* mutation.

**Results:**

Three *TNFAIP3* single nucleotide polymorphisms (SNPs; g.3033 C > T, g.3910 G > A, and g.3904 A > G) were detected in T‐ALL in the JNU dataset, and g.3033 C > T accounted for the highest proportion, reaching 60% (6/10). Interestingly, *TNFAIP3* mutation mainly occurred in adults but not pediatric patients in all three datasets (JNU, NFH, and PRJCA002270). T‐ALL patients carrying a *TNFAIP3* mutation were associated with a trend of poor overall survival (OS) (*p* = 0.092). Moreover, *TNFAIP3* mutation was also an independent factor for OS for T‐ALL patients (*p* = 0.008). Further subgroup analysis suggested that *TNFAIP3* mutation predicted poor OS for T‐ALL patients who underwent chemotherapy only (*p* < 0.001), and it was positively correlated with high risk and early T‐cell precursor ALL (ETP‐ALL) in two independent validation datasets (NFH and PRJCA002270).

**Conclusion:**

*TNFAIP3* mutation mainly occurs in adult T‐ALL patients, and it was associated with adverse clinical outcomes for T‐ALL patients; thus, it might be a biomarker for prognostic stratification.

## Graphical abstract

TNFAIP3 mutation mainly occurred in adults T‐ALL patients. TNFAIP3 mutation was associated with adverse clinical outcomes for T‐ALL patients.

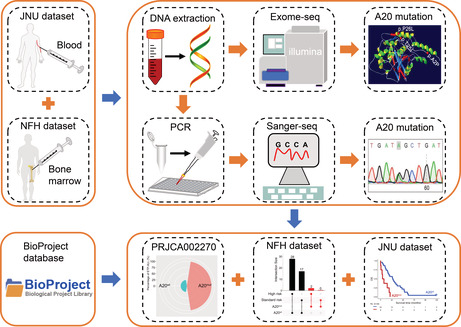

## 1 INTRODUCTION

T‐cell acute lymphoblastic leukemia (T‐ALL) is a highly aggressive leukemia with poor clinical outcome, and there is currently no effective or targeted therapy for this disease.^1^, ^2^, ^3^, ^4^, ^5^ Despite intensive chemotherapy or hematopoietic stem cell transplantation (HSCT) being used for T‐ALL, less than 50% of adults and 75% of children with T‐ALL have a 5‐year overall survival (OS), particularly for relapsed patients.^1^, ^6^, ^7^, ^8^ However, the pathogenesis of T‐ALL is relatively complex, mainly due to T‐cell receptor (TCR) rearrangement during the development and differentiation of T‐cells, which easily results in gene alterations by unstable factors.^9^, ^10^ These genetic alterations lead to malignant transformation of T‐cells, resulting in high heterogeneity of T‐ALL cells.^9^, ^10^, ^11^, ^12^ Moreover, genetic alterations exert an important role in prognostic stratification in precision therapy.^13^, ^14^, ^15^, ^16^, ^17^ Although multiple studies have been conducted to explore the role of genetic alterations combined with minimal residual disease (MRD) in risk stratification for T‐ALL, high heterogeneity has led to difficulty in accurately stratifying all patients.^14^, ^18^ Therefore, further exploration of novel biomarkers to improve risk stratification for T‐ALL patients is needed.

Tumor necrosis factor‐alpha‐inducing protein 3 (TNFAIP3), also known as A20, is an inducible protein detected in TNF‐α‐induced endothelial cells. This protein is an important negative regulator of the NF‐κB signaling pathway, which plays a critical role in the differentiation and activation of immune cells, particularly T‐cells and NKT‐cells.^19^, ^20^ TNFAIP3 plays a dual role in cancer: tumor suppressor and oncogene. Anti‐tumor effects of TNFAIP3 were shown in B cell lymphoma, colorectal carcinoma, and hepatocellular carcinoma, but TNFAIP3 acts as an oncogene in breast cancer, gastric cancer, and melanoma.^20^ In our previous publications, *TNFAIP3* mutation was significantly associated with favorable prognosis in T‐cell lymphoma (TCL) patients, and there was a low frequency of *TNFAIP3* mutation in T‐ALL.^21^, ^22^ However, prognostic analysis of *TNFAIP3* mutation in T‐ALL patients in multiple large datasets remains lacking.

In this study, we investigated the *TNFAIP3* mutation characteristics and their prognostic importance for T‐ALL patients using data from two clinical centers (JNU and NFH) and a publicly available dataset.

## 2 MATERIALS AND METHODS

### 2.1 T‐ALL patients

A total of 49 peripheral blood (PB) samples from 9 children and 40 adults were collected from de novo T‐ALL patients at JNU (Jinan University) between March 1, 2008 and April 1, 2013, and these samples were used for analysis by polymerase chain reaction (PCR) and Sanger sequencing. Corresponding clinical characteristics, including gender, age, treatment options, OS time, and event were also collected (Table 1). In addition, bone marrow (BM) samples from 54 de novo T‐ALL patients, including 7 children and 47 adults, were collected between April 12, 2011 and September 9, 2019 at NFH (Nanfang Hospital) for targeted sequencing. Clinical information, including gender, age, T‐ALL subtype, risk stratification, and treatment options, was collected. High‐risk T‐ALL is defined as one or more of the following criteria: 1. WBC: ≥100G/L; 2. Central nervous system leukemia (CNS‐L); 3. Early T‐cell precursor lymphoblastic leukemia (ETP‐ALL); 4. MLL+, t(1;19), low hyperdiploid, near triploid, or complex karyotype; 5. RAS/PTEN mutation, non‐Notch1/FBXW7 mutation; 6. MRD_1_/MRD_d15_ ≥ 1%; 7. MRD_2_/MRD_d24_ ≥ 0.1%; 8. MRD_3_/MRD_45_ ≥ 0.01%; 9. MRD (d45 to Pre‐hematopoietic stem cell transplantation) ≥ 0.01%. However, failure to meet any of the above criteria is defined as standard‐risk T‐ALL.^18^ The last follow‐up date for the adult T‐ALL patients was December 30, 2021. The endpoint of this study was OS, and the OS was defined as the time from the date of diagnosis to the date of death or last follow‐up time.^8^, ^16^, ^22^, ^23^, ^24^, ^25^ Whole‐exome sequencing data from 121 T‐ALL patients in the PRJCA002270 dataset, including 96 children, 22 adults, and 3 cases of unknown age, were downloaded from the BioProject database (https://ngdc.cncb.ac.cn/bioproject/browse/PRJCA002270).^26^

**TABLE 1:** Clinical characteristics of T‐ALL patients

Variables	JNU	NFH	PRJCA002270	*p* value
Total, *n*	49	54	121	NA
Gender, *n* (%)				0.997
Female	13 (26.5)	14 (25.9)	32 (26.4)	
Male	36 (73.5)	40 (74.1)	89 (73.6)	
Age, year, median (range)				
Children (<18 years)	7 (4–16)	15 (14–17)	8 (1–17)	< 0.001
Adult (≥18 years)	29 (18–72)	28 (18–62)	31 (18–66)	0.700
Subtype, *n* (%)				NA
ETP‐ALL	—	14 (25.9)	22 (18.2)	
Non‐ETP‐ALL	—	40 (74.1)	90 (74.4)	
Unknown	—	0 (0.0)	9 (7.4)	
Risk, *n* (%)				NA
High risk	—	37 (68.5)	—	
Standard risk	—	17 (31.5)	—	
Treatment, *n* (%)				NA
HSCT	9 (18.4)	34 (63.0)	‐	
VDCLP	25 (51.0)	20 (37.0)		
Hyper‐CVAD	6 (12.2)	0 (0.0)		
Other	9 (18.4)	0 (0.0)	—	
Sample source	PBMC	BM	—	NA

Abbreviations: BM, bone marrow; ETP‐ALL, early T‐cell precursor lymphoblastic leukemia; HSCT, hematopoietic stem cell transplantation; JNU, Jinan University; NA, not available; NFH, Nanfang Hospital; PBMC, Peripheral blood mononuclear cell; Hyper‐CVAD regimen, hyperfractionated cyclophosphamide, vincristine, doxorubicin and dexamethasone; VDCLP regimen, Vincristine, Daunorubicin, Cyclophosphamide, L‐Asparaginase, Prednisone.

### 2.2 PCR and Sanger sequencing

PB samples were collected for deoxyribonucleic acid (DNA) extraction and PCR. The detailed PCR and Sanger sequencing parameters and PCR primers for *TNFAIP3* were described in a previous publication.^21^, ^22^, ^27^

### 2.3 Targeted sequencing

Multiplex PCR primers were designed for the targeted genes using Ion Ampli‐Seq Designer (www.ampliseq.com). Nucleic acids were extracted from BM cell smears and fresh BM samples according to the instructions of DNA extraction kit (QIAGEN). The QubitdsDNA kit (Life Technologies) was used to construct the DNA library. After amplifying the target DNA, digesting the primers, adding barcode adapters, and purifying the amplified library, the Ion Library Quantitation Kit (Life Technologies) was used to quantify the library. Emulsion PCR was performed using the Ion PGMTM Template OT2 200 kit (Life Technologies), and positive template enrichment was performed using Dynabeads MyOne™ Streptavidin C1 Beads (Life Technologies) on the Ion OneTouch™ ES. Sequencing was performed on the Ion Torrent PGM using the Ion318 Chip Kit (Life Technologies). Variations, average sequencing depth, degree of reference sequence match, and filtering were analyzed using the Ion Torrent Software Suite, and the reference sequence was the human genome (GRCM7). Finally, the Sanger method was used to detect mutations in the target genes in the T‐ALL samples as a control.^21^, ^22^, ^27^

### 2.4 Construction of a TNFAIP3 protein structure model

The amino acid sequence of TNFAIP3 (NP_001257437.1) was downloaded from the NCBI database (https://ncbi.nlm.nih.gov/), and then, the protein secondary structures of TNFAIP3 were predicted by the SWISS‐MODEL database (https://www.swissmodel.expasy.org).^28^ Finally, the TNFAIP3 protein skeleton was constructed using the Swiss‐PdbViewer software (version 4.1.0),^29^ and the mutation positions in *TNFAIP3* in the NFH and PRJCA002270 datasets were indicated.

### 2.5 Statistical analysis

Statistical analysis was performed using SPSS 22.0 (IBM) and R (version 4.0.2, https://www.r‐project.org/).^30^, ^31^ Differences in subgroups in Kaplan–Meier curves were determined using the log‐rank test in the R package “survival”.^23^, ^32^, ^33^ The restricted mean survival time (RMST) was obtained by the R package “survRM2”.^24^, ^34^, ^35^ Differences between qualitative variables were compared using the chi‐square or Fisher's exact test as appropriate. The area under the curve (AUC) in the receiver operating characteristic (ROC) curve was determined by the R package “pROC”.^36^ Univariate and multivariate Cox regression models were used to confirm independent prognostic predictors.^37^ A two‐tailed *p* < 0.05 was considered statistically significant.

## 3 RESULTS

### 3.1 Mutation patterns of *TNFAIP3* in T‐ALL

**FIGURE 1:**
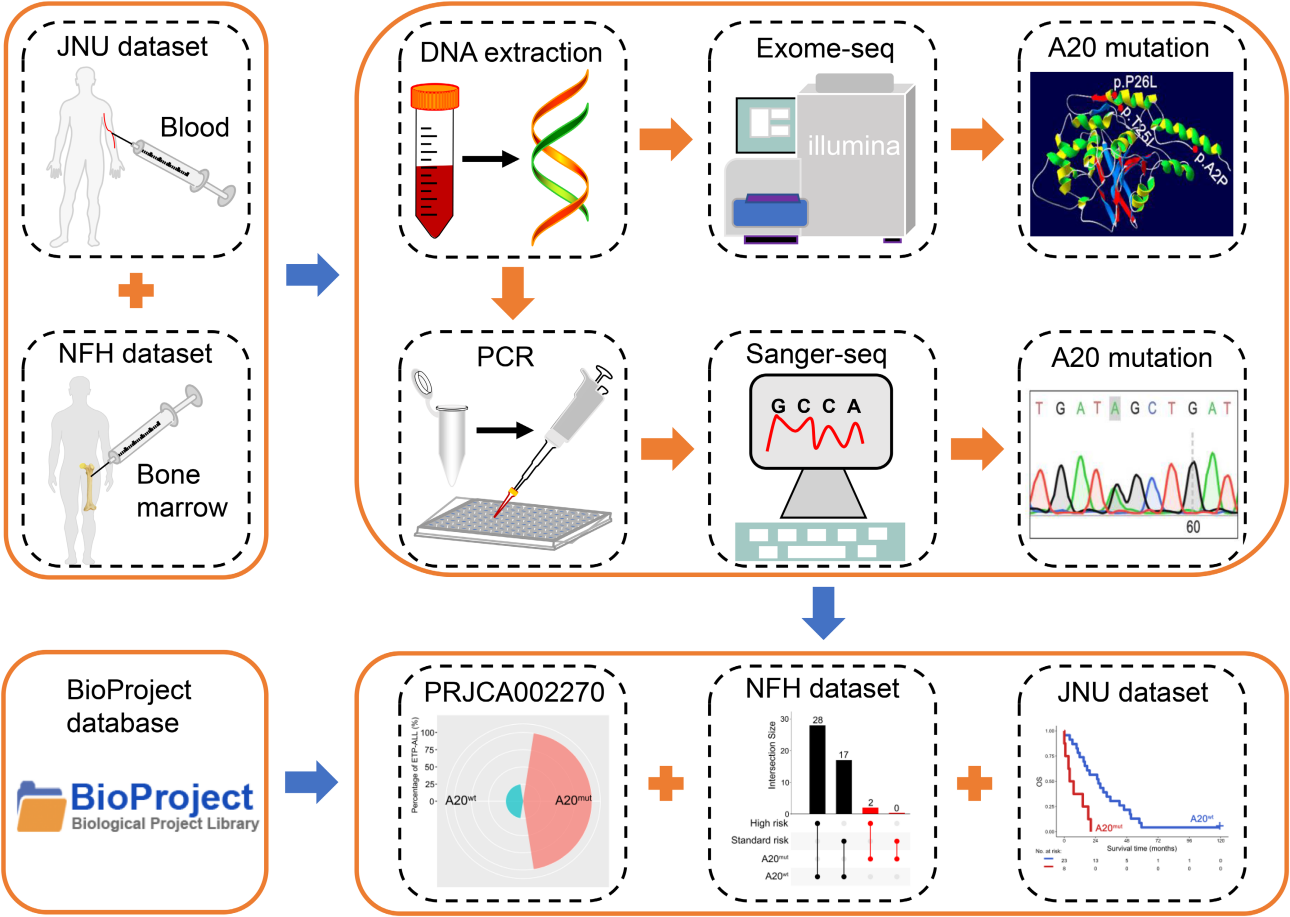
Schematic diagram of the study design. Peripheral blood (Jinan University, JNU) and bone marrow (Nanfang Hospital, NFH) samples were collected from patients with T‐cell acute lymphoblastic leukemia (T‐ALL) for DNA extraction. Then, the sites and types of *TNFAIP3* alterations were obtained by PCR and Sanger sequencing (JNU) or targeted sequencing (NFH). Finally, clinical information of T‐ALL patients was collected for prognostic analysis. In addition, whole‐exome sequencing data and clinical information from T‐ALL patients in the PRJCA002270 dataset in the BioProject database were downloaded for validation.

**FIGURE 2:**
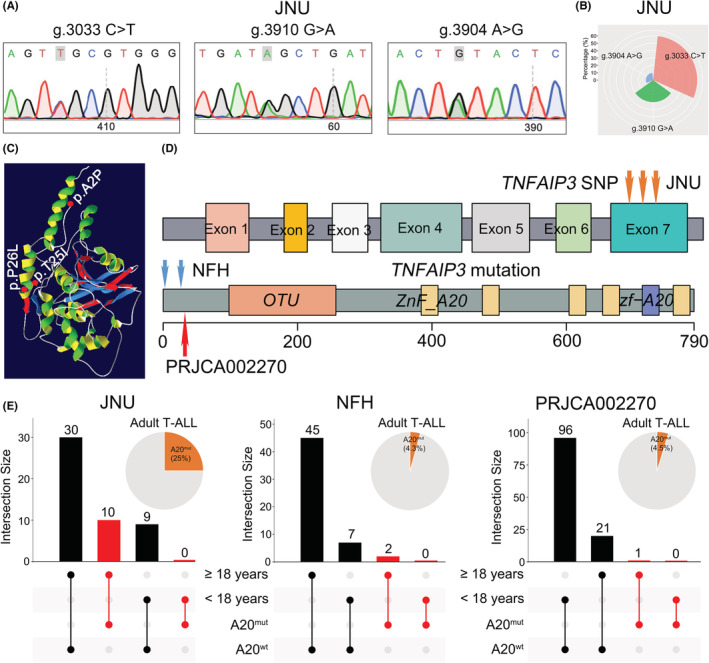
Mutation patterns of *TNFAIP3* in T‐ALL. (A) Representative *TNFAIP3* variants in the JNU dataset. (B) The proportion of the three *TNFAIP3* mutations in the JNU dataset. (C) Location of amino acid changes resulting from *TNFAIP3* mutation in the NFH and PRJCA002270 datasets. (D) Schematic of *TNFAIP3* single nucleotide polymorphism (SNP) locations in the JNU dataset (upper panel) and *TNFAIP3* mutation sites in the NFH and PRJCA002270 datasets (bottom panel). (E) Age distribution of T‐ALL patients with a *TNFAIP3* mutation in the JNU (left), NFH (middle), and PRJCA002270 (right) datasets and the frequencies of *TNFAIP3* mutation in adult patients.

Three types of *TNFAIP3* single nucleotide polymorphisms (SNPs) were detected in the JNU dataset i.e., g.3033 C > T, g.3910 G > A, and g.3904 A > G (Figures 1 and 2A). Of these, g.3033 C > T was the most common SNP in T‐ALL, accounting for 60% (6/10) (Figure 2B). These mutations resulted in amino acid changes, including g.4 G > C (p.Ala 2 Pro) and g.74 C > T (p.Thr 25 Ile) in the NFH dataset and p.Pro 26 Leu in the PRJCA002270 dataset (Figure 2C). In addition, the *TNFAIP3* SNPs were mainly located in exon 7 of *TNFAIP3* gene in the JNU dataset, and they occurred upstream of the ovarian tumor (OTU) domain in the NFH and PRJCA002270 datasets (Figure 2D). Notably, *TNFAIP3* mutations mainly occurred in adult T‐ALL patients (25%, 10/40) but not children, which was confirmed in the NFH (4.3%, 2/47) and PRJCA002270 (4.5%, 1/22) datasets (Figure 2E).

### 3.2 *TNFAIP3* mutation was associated with poor clinical outcome for T‐ALL patients who underwent chemotherapy only

**FIGURE 3:**
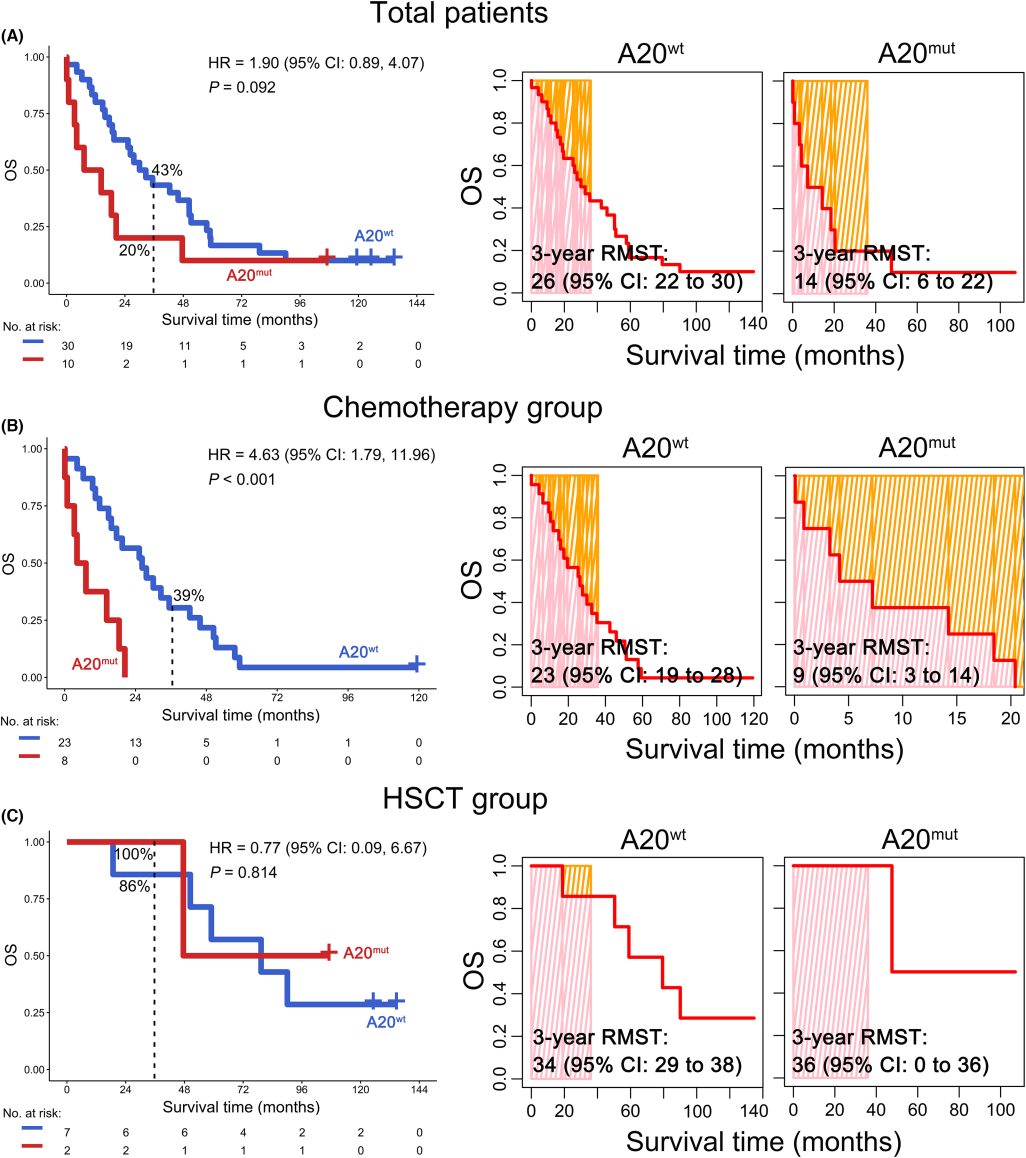
Association between *TNFAIP3* mutation and overall survival (OS) in patients with T‐ALL. (A–C) The Kaplan–Meier curves (left) and 3‐year restricted mean survival time (RMST) (right) were plotted according to *TNFAIP3* mutation status in total T‐ALL patients (A) and subgroups of patients treated with chemotherapy (B) or hematopoietic stem cell transplantation (HSCT) (C).

To elucidate the association between *TNFAIP3* mutation and OS for T‐ALL patients, prognostic analysis was performed using the JNU dataset. There was a clear trend toward poor OS for T‐ALL patients with a *TNFAIP3* mutation although the data were not statistically significant (hazard ratio (HR) = 1.90; 95% confidence interval (CI): 0.89–4.07; 3‐year OS: 20% vs. 43%; *p* = 0.092). Moreover, RMST was used to evaluate the performance of the Kaplan–Meier curve, and the 3‐year RMST for T‐ALL patients with and without *TNFAIP3* mutation was 14 (95% CI: 6–22) and 26 (95% CI: 22–30) months, respectively (Figure 3A). However, *TNFAIP3* mutation significantly predicted poor OS in T‐ALL patients (HR = 4.63; 95% CI: 1.79–11.96; 3‐year OS: 0% vs. 39%; *p* < 0.001) and a short 3‐year RMST [9 (95% CI: 3–14) vs. 23 (95% CI: 19–28) months] in the chemotherapy subgroup (Figure 3B), but it was not significantly associated with OS and 3‐year RMST in the HSCT subgroup (*p* = 0.814; Figure 3C). Importantly, when gender, age, treatment options, and *TNFAIP3* mutation status were included in univariate and multivariate Cox regression for survival analysis, the results suggested that *TNFAIP3* mutation was an independent prognostic predictor of OS for T‐ALL patients (HR = 3.14; 95% CI: 1.34–7.38; *p* = 0.008; Table 2).

**TABLE 2:** Univariate and multivariate Cox regression analyses of *TNFAIP3* mutation in adult T‐cell acute lymphoblastic leukemia (T‐ALL) patients in the Jinan University (JNU) dataset

Variable	Univariate Cox		Multivariate Cox	
	HR (95% CI)	*p* value	HR (95% CI)	*p* value
TNFAIP3				
wt	Reference		Reference	
mut	1.90 (0.89, 4.07)	0.0	3.14 (1.34, 7.38)	0.008
Gender				
Female	Reference		Reference	
Male	0.64 (0.31, 1.31)	0.225	0.75 (0.36, 1.56)	0.439
Treatment				
Chemotherapy	Reference		Reference	
HSCT	0.22 (0.09, 0.56)	0.001	0.18 (0.06, 0.48)	0.001
Age, years	1.02 (0.99, 1.04)	0.145	1.00 (0.98, 1.03)	0.751

Abbreviations: CI, confidence interval; HR, hazard ratio; HSCT, hematopoietic stem cell transplantation; mut, mutation; wt, wildtype.

**FIGURE 4:**
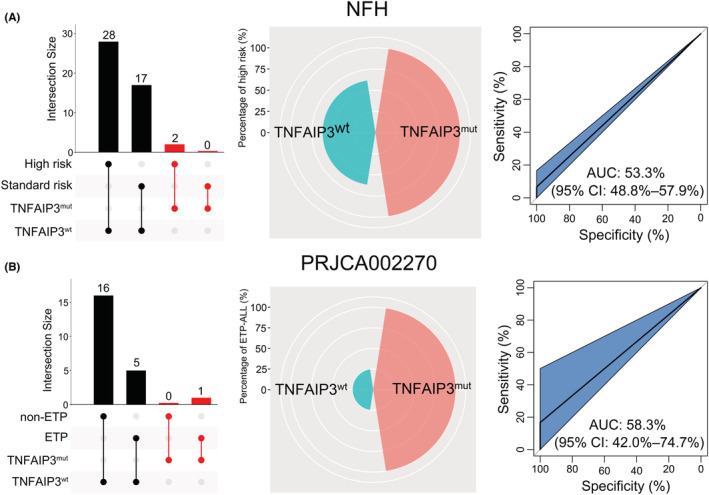
Relationship between *TNFAIP3* mutation and risk stratification or T‐ALL subtype. (A, B) *TNFAIP3* mutation association with risk stratification and T‐ALL subtype in the NFH (A) and PRJCA002270 (B) datasets. The number of *TNFAIP3* mutations in the high vs. standard risk and early T‐cell precursor ALL (ETP‐ALL) vs. non‐ETP‐ALL subgroups (left). The proportion of high‐risk and ETP‐ALL patients with or without a *TNFAIP3* mutation (middle). Receiver Operating Characteristic (ROC) curves were used to assess the sensitivity of *TNFAIP3* mutation for high risk and ETP‐ALL (right).

To validate the findings in the JNU dataset, the targeted sequencing data from the NFH and PRJCA002270 datasets were analyzed. The results indicated that two high‐risk patients and one early T‐cell precursor ALL (ETP‐ALL) patient had *TNFAIP3* mutation (Figure 4A,B, left panels). Furthermore, the proportion of high‐risk or ETP‐ALL patients in the *TNFAIP3* mutation subgroup was higher than that in the *TNFAIP3* wild‐type subgroup (high risk: 100% vs. 62%; ETP‐ALL: 100% vs. 25%) (Figure 4A,B, middle panels). Additionally, an ROC curve suggested that *TNFAIP3* mutation had weak sensitivity for high‐risk T‐ALL (AUC = 53.3%, 95% CI: 48.8%–57.9%) and ETP‐ALL (AUC = 58.3%, 95% CI: 42.0%–74.7%; Figure 4A,B, right panels). These findings suggest that TNFAIP3 mutation is significantly associated with poor clinical outcome for T‐ALL patients.

## 4 DISCUSSION

TNFAIP3 is a ubiquitin‐editing enzyme with ubiquitin ligase activity and deubiquitinase (DUB) activity. The amino terminus of TNFAIP3 contains an ovarian tumor (OUT) domain, which has DUB activity, and the carboxy terminus contains seven zinc finger (ZnF) domains that mediate ubiquitin ligase and ubiquitin‐binding activity.^19^, ^20^ The structure of TNFAIP3 could explain its dual role in cancer.^20^, ^38^, ^39^, ^40^ For example, TNFAIP3 can negatively regulate the NF‐κB signaling pathway through cooperation of the OTU and ZnF4 domains.^20^, ^41^ In this study, *TNFAIP3* mutations were mainly located upstream of the OTU domain, which might affect the cooperation of the OTU and ZnF domains in deubiquitination and ubiquitination, resulting in poor prognoses for T‐ALL patients. These findings are consistent with multiple studies on B‐cell lymphoma.^42^, ^43^, ^44^ However, in contrast to the results in T‐ALL, our previous publication suggested that *TNFAIP3* mutation predicts favorable OS for TCL patients.^22^ The reasons for the survival benefit may be the heterogeneity in these two types of T‐cell malignancies and the differences in treatment options. Whether there are different roles for TNFAIP3 in TCL and T‐ALL needs to be explored in the future. Notably, statistical significance was not achieved for OS between patients with and without *TNFAIP3* mutation in all of the adult T‐ALL patients, which might be due to the effects of HSCT treatment. However, when we compared the different therapeutic approaches for T‐ALL, *TNFAIP3* mutation significantly predicted poor OS for adult T‐ALL patients treated with chemotherapy, but it was not associated with OS for patients treated with HSCT; thus, it is thought that HSCT could overcome these poor genetic alterations. HSCT has been well established to cure and improve the prognosis of patients with acute leukemia, but its effects may be related to different adverse genetics.^45^, ^46^, ^47^ Importantly, the poor prognostic impact of some mutations was lost when the patients received HSCT, implying that HSCT might overcome the poor risk of acute leukemia patients with these mutations.^48^, ^49^ It would be more convincing if we could validate these results with a database of patients or another set of T‐ALL samples. Thus, we also attempted to find another publicly available dataset to validate our results. Unfortunately, there are no datasets containing OS information and *TNFAIP3* mutation status for further analysis and validation. In addition, increasing evidence indicates that high‐risk stratification is significantly associated with adverse outcomes for T‐ALL patients compared to standard‐risk groups.^18^

ETP‐ALL is a T‐ALL subtype characterized by a lack of mature T‐cell markers and the expression of stem and myeloid‐lineage genes, and this subtype has poor prognosis compared with non‐ETP‐ALL patients.^50^, ^51^ Therefore, we validated our OS findings by analyzing the association between *TNFAIP3* mutation and risk stratification for this T‐ALL subtype. Our results clearly demonstrated that *TNFAIP3* mutation is positively correlated with the high‐risk stratification and ETP‐ALL, suggesting that *TNFAIP3* mutation might be associated with adverse clinical outcomes in T‐ALL patients. However, more T‐ALL samples are needed to validate these findings in the future.

Although pediatric and adult T‐ALL are both heterogeneous diseases caused by accumulation of genetic alterations in progenitor T‐cells, there are differences in the mutation patterns between pediatric and adult patients.^26^, ^52^ Importantly, exome sequencing has identified recurrent genetic alterations in T‐ALL. For example, NOTCH1 mutations are present in greater than 60% of T‐ALL cases, and activation of oncogenic transcription factors, which often results from rearrangement to the TCR locus, is also a biomarker for T‐ALL.^52^, ^53^ However, some genetic alterations remain unidentified between pediatric and adult patients. In this study, *TNFAIP3* mutation was mainly localized in adult but not pediatric patients, suggesting that *TNFAIP3* mutation might be a biomarker for adult T‐ALL patients. Of course, this finding requires evaluation of the sensitivity and specificity in detecting *TNFAIP3* mutations in the diagnosis of adult T‐ALL patients using greater numbers of T‐ALL samples in the future. In addition, whether *TNFAIP3* mutations are accompanied by other poor prognostic factors in predicting adverse clinical outcomes, such as co‐mutations, chromosomal abnormalities, and abnormal gene expression, needs to be further explored in the future.

However, firstly, the limitation of this study is its small sample size in our clinical center, which may lead to statistical bias for *TNFAIP3* mutation in predicting the clinical outcomes of T‐ALL patients. Furthermore, OS data were lacking in the NFH dataset to validate the prognostic importance of *TNFAIP3* mutation for T‐ALL patients. Moreover, this study focus on the effects of TNFAIP3 mutation for OS, further studies may include the effects of TNFAIP3 mutation on the disease relapse, treatment‐related mortality (TRM), and leukemia‐free survival (LFS), etc.

## 5 CONCLUSION

We for the first time characterize *TNFAIP3* mutation patterns and demonstrate that they mainly occur in adult T‐ALL patients and suggest that *TNFAIP3* mutation might be a predictor of adverse clinical outcomes in T‐ALL patients, which might complement current prognostic stratification for T‐ALL patients.

## AUTHORS' CONTRIBUTIONS

CTC and LLZ interpreted the data, performed the experiments, and wrote the manuscript. LHZ helped to interpret the data. GXL, LW, and HSZ diagnosed and treated the patients and provided clinical information. CWZ edited and reviewed the manuscript. YQL contributed to the concept development and study design and edited the manuscript. All authors read and approved the final manuscript.

## FUNDING INFORMATION

This work was supported by grants from the Guangdong Science and Technology Project (No. 2020A0505100042), the Intergovernmental International Cooperation on Scientific and Technological Innovation Project of Chinese Ministry of Science and Technology (No. 2017YFE0131600), and Research Grant of Key Laboratory of Regenerative Medicine, Ministry of Education, Jinan University (No. ZSYXM202204).

## CONFLICT OF INTEREST

All authors declare no conflict of interest.

## ETHICS APPROVAL

This study was performed according to the Declaration of Helsinki principles and approved by the Ethics Committee of Jinan University and Affiliated Nanfang Hospital of Southern Medical University. All participants provided written informed consent.

## Data Availability

The PRJCA002270 dataset used in this study was acquired from the BioProject database (https://ngdc.cncb.ac.cn/bioproject/browse/PRJCA002270). The datasets used and/or analyzed during this study are available from the corresponding author upon reasonable request.
